# The effect of vitamin D supplementation on serum total 25(OH) levels and biochemical markers of skeletal muscles in runners

**DOI:** 10.1186/s12970-020-00347-8

**Published:** 2020-04-09

**Authors:** Aleksandra Żebrowska, Ewa Sadowska-Krępa, Arkadiusz Stanula, Zbigniew Waśkiewicz, Olga Łakomy, Eduard Bezuglov, Pantelis T. Nikolaidis, Thomas Rosemann, Beat Knechtle

**Affiliations:** 1 Institute of Sport Sciences, Academy of Physical Education in Katowice, Mikołowska Street 72a, 40-065 Katowice, Poland; 2 Department of Sport Medicine and Medical Rehabilitation, Sechenov First Moscow State Medical University (Sechenov University), 119435 Moscow, Russia; 3 Exercise Physiology Laboratory, Nikaia, Greece; 4 Institute of Primary Care, University of Zurich, Zurich, Switzerland; 5 Medbase St. Gallen Am Vadianplatz, Vadianstrasse 26, 9001 St. Gallen, Switzerland

**Keywords:** Vitamin D, Muscle biomarkers, Eccentric exercise, Fatigue, Marathon

## Abstract

**Background:**

The beneficial adaptation of skeletal muscle function to strenuous exercise is partially attributable to the improvement of vitamin D status. The present study aimed to evaluate the effects of a 3-week vitamin D supplementation on serum 25(OH)D levels and skeletal muscle biomarkers (i.e. troponin, myoglobin, creatine kinase and lactic dehydrogenase) of endurance runners.

**Methods:**

A double-blind placebo-controlled study design was used and vitamin D supplementation was compared to a non-treatment control group. Twenty-four runners, competitors of the ultra-marathons held during the National Running Championships, were randomly assigned into two groups supplemented with the dose of 2000 IU vitamin D or placebo for three weeks. All subjects participated in three exercise protocols: (a) incremental exercise test (to determine the maximum oxygen uptake and the intensity of eccentric exercise), (b) eccentric exercise before and (c) after two dietary protocols. Venous blood samples were drawn at rest, immediately after the exercise and after 1 h and 24 h of recovery in order to estimate serum 25(OH)D levels, skeletal muscle biomarkers, proinflammatory cytokines and tumor necrosis factor-alpha (TNF-α) levels. A two-way ANOVA was used to test main effects and their interactions and Pearson correlation coefficients were analyzed to determine the effects of inter-variable relationships.

**Results:**

Significant differences between pre- and post-intervention in baseline 25(OH)D levels were observed (34.9 ± 4.7 versus 40.3 ± 4.9 ng/ml*, p =* 0.02) in supplemented group. A higher post intervention 25(OH)D level was observed after vitamin D diet compared to placebo (40.3 ± 4.9 versus 31.8 ± 4.2 ng/mL, respectively; *p <* 0.05). The vitamin D supplementation decreased post-exercise (TN max) and 1 h post-exercise troponin (*p =* 0.004*, p* = 0.03, respectively), 1 h post-exercise myoglobin concentration (*p* = 0.01) and TNF-α levels(*p* < 0.03). 24 h post exercise creatine kinase activity was significantly lower in supplemented group compared to placebo (p < 0.05). A negative correlation was observed between post exercise 25(OH)D levels and myoglobin levels (*r* = − 0.57*; p* = 0.05), and 25(OH)D levels and TNFα (r = − 0.58; *p* = 0.05) in vitamin D supplemented group.

**Conclusions:**

Three weeks of vitamin D supplementation had a positive effect on serum 25(OH)D levels in endurance trained runners and it caused a marked decrease in post-exercise biomarkers levels. We concluded that vitamin D supplementation might play an important role in prevention of skeletal muscle injuries following exercise with eccentric muscle contraction in athletes.

## Introduction

Endurance training has been associated with adaptive changes in skeletal muscle, such as an ability to use oxygen to generate energy for muscle work, a decrease in oxygen demand for the same level of external work performed [1], and a modification in markers of muscle damage and inflammation [2]. In a recent study, a prevalence of vitamin D deficiency in extreme endurance athletes, and an association between a delayed physical performance and a deficiency in vitamin D were observed during regular training [2–4]. These physiological responses in skeletal muscles were influenced by exercise-induced mechanisms and were probably affected by the nutritional athletic status and a limitation of sun exposure [5–7].

Neuromuscular consequences of mountain marathon running has been shown to induce changes in neuromuscular function [8] and blood markers of muscle damage and inflammation [7, 9–11]. The potential mechanisms - through which function of the muscular system might be beneficially modified in response to extreme repeated exercise stress - included improvement of vitamin D status [12]. Several studies supported the theory that functional responses in skeletal muscle were influenced by mechanisms that could be affected by biological effects of an active form of vitamin D and its ability to bind with the membrane and nuclear vitamin D receptors (VDRs) [13, 14]. Besides the importance of vitamin D, especially 25(OH)D (serum 25-hydroxy vitamin D), in the regulation of bones and calcium homeostasis, it was also involved in skeletal muscle performance and in exercise-induced inflammatory processes, neurological functions and cardiovascular health [15–17].

It should be noted that muscle power and force in marathon runners were linked with vitamin D levels [17]. The deficiency in vitamin D increased the risk of muscle myopathy, and impaired cross-bridge formation leading to muscle weakness and fatigue [18–20]. Due to the higher levels of biomarkers of muscle injury and reduction of total antioxidant capacity and muscle function in response to extreme exercise training, strategies should be developed to maintain an optimal vitamin D level in response to its exercise-induced deficiency. It has been hypothesized that a higher exposure to vitamin D-producing ultraviolet light and serum 25(OH)D levels above the normal reference range (up to 50 ng^/^mL) could be associated with beneficial adaptations in skeletal muscle consisting of an enhanced aerobic performance, both force and power production and a decreased recovery time from training [21].

The physiological consequence of intense physical training in response to vitamin D supplementation induced by activation of the serum 25(OH)D status depended on the dosages exceeding the recommendations for vitamin D [22–25]. In elite rowers, maximal oxygen uptake increased significantly in response to supplementation with 6000 IU/day of vitamin D during 8-weeks training, whereas, the dosage of 4000 IU/day for 35 days of vitamin D improved the recovery by the attenuation of the inflammation processes in moderately physically active adults [26]. Positive effects of supplementation (8 weeks of 5000 IU/day of vitamin D) and increases in force and power production in professional soccer players were also observed [25].

 However, optimal vitamin D dosage and serum levels needed for athletic performance and recovery have been controversial [21, 26]. A dosage of 600–800 IU/day and 1000 IU/day of vitamin D might not be sufficient for optimal levels of vitamin D, nor prevent a decline in serum 25(OH)D in response to intense exercise training [22]. There was evidence suggesting that dietary supplementation with 2000 to 5000 IU/day of vitamin D had a positive impact on bone health and skeletal muscle function [24]. However, it was not specified what dose of vitamin D was sufficient to ameliorate muscle damage and could be effective for accelerating muscle regeneration after intense effort with an eccentric work component [27, 28].

Participation in marathon and ultra-marathon races is becoming an increasingly popular activity, which is encouraged by an increasing number of running events being organized each year. Hence, a number of investigations have been conducted to determine the risk factors of skeletal muscle injury in long-term runners [2, 9, 29]. Considering that fact, there are still, at present, no official recommendations for the treatment of muscle fatigue. Non-specific treatments with a higher vitamin D usage have been used clinically or experimentally, and have shown some positive effects.

Therefore, it seemed important to investigate the association between recommended low vitamin D dosage and an early identification of increased muscle fatigue risk. In previous studies on the assessment of muscle dysfunction, the conventional biomarkers (e.g.*,* Tn, CK, myoglobin, LDH) have been analyzed [30, 31]. These markers had different release times and different times of reaching maximal concentrations [9, 11, 32]. It has been hypothesized that exercise-induced lower muscle biomarker secretion may depend on increased serum 25(OH)D levels and these vitamin levels might be used for early detection of greater muscle resistance to eccentric-induced muscle fatigue [31]. There are limited data regarding the effect of lower dosages of vitamin D supplementation on muscle function and optimization of recovery mechanisms of elite ultramarathon runners. It was also hypothesized that higher serum 25(OH)D levels in response to low dosage of vitamin D supplementation might improve this function via the stimulation of 25(OH)D production and release [26]. To verify this, the relationships between eccentric exercise-induced muscle biomarker levels, as measured by troponin, myoglobin concentrations and creatine kinase and lactic dehydrogenase activity and 25(OH)D levels in response to vitamin D supplementation in marathon runners were examined.

## Material and methods

### Ethical approval

The experiment was approved by the Ethics Committee of the Academy of Physical Education in Katowice (Ethics Committee decision KBN 3.2016) and conformed to the standards set by the Declaration of Helsinki.

### Subjects

Twenty-four male ultramarathon Caucasian runners who were endurance-trained for about seven years were randomly assigned to either dietary protocol (i.e. placebo or the vitamin D supplementation, a double-blind placebo-controlled study). Study members were recruited from all the competitors of the ultra-marathons held during the Polish Running Championships. The inclusion criteria were participation in at least five marathons and written informed consent to take part in the study.

In this double-blind study, participants were randomly assigned, with a 1:1 assignment, to either the vitamin D supplement group (EXP) or the placebo control group (CON) (utilizing computer-generated random numbers): 12 subjects were assigned to the EXP and 12 subjects were assigned to the CON. This study design (two groups and two exercise bouts) allowed to exclude the influence of environmental factors such ultraviolet exposure, nutritional and training statues on post-intervention 25(OH)D levels. Age, height, body mass, body mass index (BMI), body composition and the training status of the subjects the participants are presented in Table 1.

**Table 1:** Subject characteristics (mean ± SD)

Variables	EXP *n* = 12	CON *n* = 12	*p*-value
Age (years)	33.7 ± 7.5	35.9 ± 5.3	0.44
Body mass (kg)	74.7 ± 10.6	75.3 ± 8.6	0.89
Body Height (cm)	176.8 ± 6.0	178.2 ± 6.8	0.62
BMI (kg/m^2^)	23.8 ± 2.2	23.7 ± 2.1	0.91
FAT (%)	13.7 ± 3.3	13.5 ± 4.4	0.91
SMM (kg)	36.5 ± 5.1	36.9 ± 4.5	0.85
TBW (L)	47.2 ± 6.4	47.5 ± 5.4	0.91
VO_2_peak (mL/kg/min)	54.5 ± 9.4	58.1 ± 7.4	0.33
Peak power (Watt)	321.5 ± 77.9	351.4 ± 68.3	0.35
HR max (b/min)	181.0 ± 11.0	186.0 ± 9.0	0.26

*BMI* body mass index, *FAT* percent of body fat, *SMM* skeletal muscle mass, *TBW* total body water, *VO*_*2*_*peak* peak oxygen uptake, *HR max* heart rate maximum

Athletes were interviewed by the research staff using a survey with questions regarding nutrition, previous supplementation and their training history. Mean energy supply with diet, mean daily fat, carbohydrate, protein and vitamin D intake were comparable in the supplemented group and placebo group (Table 2). Biochemical measurements of pre- intervention 25(OH)D levels in runners indicated that serum levels of 25(OH)D did not differ between the groups (Table 3). None of the participants had a vitamin D deficiency or toxicity (< 20 ng/mL or > 100 ng/mL). All subjects reported that they were not taking any medication that could affect the 25(OH)D status. All subjects participated in the study during the pre-season period (February and March) to minimize variability due to ultraviolet exposure. The melanin pigmentary system was not different between the groups (10–14 von Luschan’s chromatic scale). During the study they were instructed to abstain from strenuous exercise. No caffeine, supplements, or alcohol were permitted during the experiment. Three weeks prior to the study all participants were put on a mixed diet (Table 2). The composition of the diet was calculated using dedicated software for each subject (Dietus, B.U.I. InFit. Warsaw, Poland). The diet was continued with vitamin D or placebo administration. To ensure that participants adhered to the dietary regimen, they had to keep daily food intake logs which were inspected during the weekly, obligatory visits in the laboratory. We supplemented our subjects for 3 weeks and before each diet protocol, the biochemical variables and physiological variables were analyzed.

**Table 2:** Mean energy supply with diet, mean daily fat, carbohydrate, protein and vitamin D intake in the supplemented group and placebo group (mean, SD)

Variables	*EXP* (*n* = 12)	*CON* (*n* = 12)	*p*-value
Energy [kcal/kg/day]	29.6 *±* 3.0	28.0 *±* 2.0	0.23
Fat intake [%]	31.7 *±* 9.6	30.8 *±* 8.3	0.96
Carbohydrate intake [%]	46.1 *±* 6.6	46.7 *±* 8.5	0.90
Protein intake [%]	22.8 *±* 5.4	22.4 *±* 3.3	0.73
Vitamin D [μg/day]	7.8 *±* 7.1	8.4 *±* 7.3	0.97

**Table 3:** Serum 25(OH)D levels and biochemical markers of muscle damaged of the subjects

Variables	Group	Pre-Intervention	Post-Intervention	Effect size	*p*-value	Mean difference (%)	CI −95%	CI + 95%
25(OH)rest [ng/ml]	EXP	34.9 ± 4.7	40.3 ± 4.9*	0.69 / Moderate	**0.02**	−3.9 (− 10.7%)	−7.03	− 0.72
	CON	33.9 ± 4.8	**31.8 ± 4.2**	0.46 / Small	0.13	2.1 (6.2%)	−0.68	4.88
25(OH)max [ng/ml]	EXP	36.5 ± 3.3	44.9 ± 4.9	1.64 / Large	**< 0.001**	−7.7 (−20.9%)	− 11.14	−4.30
	CON	34.7 ± 8.1	39.2 ± 7.6	0.57 / Small	0.13	−4.5 (−13.0%)	−10.57	1.55
25(OH)D1 h [ng/ml]	EXP	40.0 ± 8.8**	45.5 ± 4.7*	0.52 / Small	0.22	−3.9 (−9.4%)	− 10.44	2.70
	CON	**33.3 ± 3.4**	**38.5 ± 9.7**	0.71 / Moderate	**0.04**	−5.2 (−15.6%)	−10.11	−0.29
25(OH)D24h [ng/ml]	EXP	36.2 ± 6.2*	41.2 ± 5.0*	0.76 / Moderate	**0.04**	−4.6 (−12.5%)	−9.00	−0.14
	CON	**30.0 ± 6.4**	**35.7 ± 6.9**	0.87 / Moderate	**0.02**	−5.8 (− 19.4%)	−10.63	−0.99
TN rest [ng/ml]	EXP	2.9 ± 1.9**	2.0 ± 1.6*	0.77 / Moderate	0.12	1.7 (45.3%)	−0.51	3.82
	CON	**7.2 ± 2.0**	**5.6 ± 4.2**	0.61 / Moderate	0.20	3.0 (35.2%)	−1.82	7.85
TN max [ng/ml]	EXP	5.1 ± 1.7***	2.7 ± 1.6***	1.44 / Large	**0.004**	2.4 (47.4%)	0.94	3.94
	CON	**8.9 ± 6.3**	**5.3 ± 4.1**	1.38 / Large	**0.02**	7.6 (59.3%)	1.73	13.56
TN 1 h [ng/ml]	EXP	4.9 ± 2.0	2.9 ± 2.0	0.98 / Moderate	**0.03**	2.0 (40.8%)	0.18	3.84
	CON	4.4 ± 3.2	4.7 ± 2.4	0.09 / Trivial	0.82	−0.3 (− 5.8%)	−2.63	2.12
TN 24 h [ng/ml]	EXP	6.3 ± 3.7	3.7 ± 1.2	0.82 / Moderate	0.08	2.6 (40.7%)	−0.33	5.47
	CON	4.7 ± 1.2	3.1 ± 1.2	0.47 / Small	0.28	1.6 (33.5%)	−1.49	4.65
MB rest [ng/ml]	EXP	44.7 ± 23.1	40.6 ± 17.6	0.19 / Trivial	0.71	4.1 (9.2%)	−19.36	27.58
	CON	44.4 ± 11.8	37.1 ± 21.8	0.39 / Small	0.43	7.2 (16.3%)	−12.11	26.54
MB max [ng/ml]	EXP	73.9 ± 32.0	58.7 ± 27.6	0.49 / Small	0.16	15.2 (20.6%)	−7.12	37.52
	CON	93.4 ± 33.1	73.5 ± 43.7	0.49 / Small	0.29	19.9 (21.3%)	−19.84	59.67
MB 1 h [ng/ml]	EXP	173.6 ± 104.5	92.6 ± 48.9	0.96 / Moderate	**0.01**	81.5 (47.0%)	24.69	138.35
	CON	102.6 ± 59.5	83.9 ± 50.0	0.32 / Small	0.47	18.6 (18.2%)	−36.60	73.82
MB 24 h [ng/ml]	EXP	93.2 ± 56.2	59.5 ± 37.8	0.67 / Moderate	0.15	33.7 (36.2%)	−14.17	81.60
	CON	98.3 ± 26.7	93.0 ± 50.7	0.13 / Trivial	0.72	5.3 (5.4%)	−26.31	37.01
CK rest [U/l]	EXP	151.0 ± 59.5*	166.4 ± 100.0	0.19 / Trivial	0.58	−15.4 (− 10.2%)	−75.57	44.72
	CON	**234.2 ± 88.9**	248.4 ± 179.0	0.1 / Trivial	0.81	−14.1 (−6.0%)	− 143.10	114.84
CK max [U/l]	EXP	226.1 ± 141.0	212.7 ± 112.0	0.1 / Trivial	0.78	13.4 (5.9%)	−87.82	114.67
	CON	276.2 ± 118.2	286.6 ± 191.5	0.06 / Trivial	0.87	−10.4 (−3.8%)	− 152.70	131.84
CK 1 h [U/l]	EXP	248.0 ± 161.8	214.3 ± 109.0	0.23 / Small	0.46	33.7 (13.6%)	−63.22	130.62
	CON	276.8 ± 122.3	213.2 ± 113.4	0.52 / Small	0.16	63.5 (22.9%)	−29.34	156.39
CK 24 h [U/l]	EXP	361.3 ± 238.9	243.3 ± 91.5*	0.55 / Small	0.20	97.7 (28.1%)	−59.56	255.03
	CON	434.3 ± 143.9	**332.0 ± 155.6**	1.17 / Moderate	**0.05**	143.1 (32.9%)	64.74	221.49
LDH rest [U/l]	EXP	337.1 ± 73.5	333.1 ± 80.5	0.05 / Trivial	0.82	4.0 (1.2%)	−34.60	42.60
	CON	339.4 ± 47.8	333.1 ± 60.1	0.11 / Trivial	0.69	6.2 (1.8%)	−27.30	39.74
LDH max [U/l]	EXP	400.5 ± 108.0	395.9 ± 68.6	0.18 / Trivial	0.54	17.5 (4.4%)	−44.25	79.36
	CON	401.4 ± 63.8	413.5 ± 79.6	0.16 / Trivial	0.33	−12.1 (−3.0%)	−38.25	14.04
LDH 1 h [U/l]	EXP	361.4 ± 87.8	354.2 ± 69.4	0.21 / Small	0.37	18.3 (5.1%)	−25.25	61.88
	CON	355.0 ± 44.9	368.6 ± 72.2	0.22 / Small	0.35	−13.6 (−3.8%)	−43.99	16.86
LDH 24 h [U/l]	EXP	344.9 ± 75.5	313.5 ± 66.6	0.02 / Trivial	0.94	−1.8 (−0.5%)	−51.33	47.68
	CON	339.1 ± 56.8	321.1 ± 31.1	0.07 / Trivial	0.74	4.1 (1.2%)	−21.87	29.99
TNFα rest [pg/ml]	EXP	9.7 ± 5.7	5.6 ± 2.6	0.88 / Moderate	0.09	4.1 (42.2%)	−0.75	8.93
	CON	13.7 ± 7.4	12.5 ± 4.4	0.19 / Trivial	0.65	1.2 (8.9%)	−4.52	6.96
TNFα max [pg/ml]	EXP	23.9 ± 15.2	10.5 ± 4.6	1.14 / Moderate	**0.01**	13.4 (56.2%)	3.48	23.40
	CON	22.9 ± 13.7	22.7 ± 17.4	0.01 / Trivial	0.97	0.2 (0.8%)	−12.6	12.98
TNF α 1 h [pg/ml]	EXP	21.9 ± 16.8	8.4 ± 3.7	1.06 / Moderate	**0.03**	13.5 (61.7%)	1.74	25.26
	CON	18.7 ± 11.4	21.3 ± 12.2	0.21 / Small	0.65	−2.6 (−13.9%)	−14.91	9.69
TNF α 24 h [pg/ml]	EXP	19.8 ± 14.2	11.6 ± 5.7	0.73 / Moderate	**0.02**	8.2 (41.6%)	1.55	14.94
	CON	13.9 ± 6.7	13.7 ± 7.3	0.02 / Trivial	0.95	0.1 (1.1%)	−5.56	5.87
IL-6 rest [pg/ml]	EXP	1.4 ± 1.3	1.9 ± 1.8	0.25 / Small	0.23	−0.3 (−18.7%)	−0.74	0.20
	CON	1.5 ± 1.3	2.1 ± 1.6	0.49 / Small	0.10	−0.6 (−44.1%)	−1.42	0.15
IL-6 max [pg/ml]	EXP	2.0 ± 1.9	1.7 ± 1.0	0.29 / Small	0.39	0.33 (16.1%)	−0.49	1.15
	CON	2.7 ± 1.5	2.5 ± 2.3	0.01 / Trivial	0.98	−0.01 (− 0.4%)	−1.18	1.16
IL-6 1 h [pg/ml]	EXP	2.7 ± 2.3	2.3 ± 1.3	0.22 / Small	0.59	0.39 (14.4%)	−1.14	1.92
	CON	3.1 ± 2.0	3.0 ± 2.0	0.02 / Trivial	0.95	−0.04 (−1.5%)	−1.40	1.32
IL-6 24 h [pg/ml]	EXP	1.8 ± 1.2	1.0 ± 0.9**	0.72 / Moderate	0.11	0.79 (43.5%)	−0.20	1.77
	CON	2.0 ± 1.2	**2.4 ± 1.6**	0.26 / Small	0.46	−0.3 (−14.9%)	−1.17	0.57

* *p* < 0.05; ** *p* < 0.01; *** *p* < 0.001 significant differences between vitamin D supplemented group (EXP) and placebo control group (CON)

### Study design

All subjects participated in the following experiment consisting of three protocols: (1) performance test to determine the current training level and intensity of continuous eccentric exercise (downhill running), (2) continuous eccentric exercise before intervention, and (3) continuous eccentric exercise post intervention. The first and second laboratory protocols were separated by at least seven days to prevent any possible interference on the subjects’ exercise abilities or fatigue.

### Supplementation procedure

All clinical data, including biochemical parameters and exercise examination, were obtained after an overnight fast at baseline and in response to both experimental protocols. Following these measurements, blood samples were taken through a peripheral catheter inserted into the antecubital vein. After initial testing, the vitamin D supplemented group (EXP) received 50 μg (2 × 1000 IU/day) of vitamin D as cholecalciferol. The control group (CON) received a placebo in the form of gelatin capsules (1.3 g lactose monohydrate). Participants were instructed to take the capsules with meals twice daily for a total of 3 weeks.

### Exercise protocols

#### Peak aerobic exercise capacity (VO_2peak_)

At the baseline, before treatment protocol (supplementation or placebo), all subjects performed a standard incremental treadmill exercise test (LE 200 treadmill, Jaeger, Frankfurt, Germany) to measure their individual aerobic performance (peak oxygen uptake, VO_2_peak). The test started with a 3-min warm-up at 6 km/h and 0% inclination; the intensity was then increased by 2 km/h every 3 min up to 12 km/h and then the intensity was increased and inclination by 2.5% up to maximal exercise intensity or volitional fatigue. Heart rate (HR) (PE-3000 Sport-Tester, Polar Inc., Kempele, Finland) and systolic and diastolic blood pressure (SBP/DBP) were measured (HEM-907 XL, Omron Corporation, Kyoto, Japan) before and immediately after the test. Pulmonary ventilation (VE), oxygen uptake (VO_2_), and carbon dioxide output (CO_2_) were measured continuously from the 6 min prior to exercise test and throughout each stage of the exercise test using the Oxycon Apparatus (CareFusion Germany 234 GMBH, Hoechberg Jaeger, Germany). Maximal exercise intensity (peak power, Ppeak) was calculated as a function of the body weight, treadmill speed and gradient and exercise duration (Watt). Criteria for termination of VO_2_peak were voluntary exhaustion, respiratory ratio equal to or exceeding 1.15 (RER ≥1.1), a VO_2_ plateau and blood lactate concentration ≥ 8.0 mmol/L. Physiological characteristics of the participants are presented in Table 1.

#### Eccentric muscle damage exercise

All subjects performed a 30-min downhill running test with an eccentric type of work (ExE) and intensity of their individual 70% VO_2_peak and treadmill 16% declination based on a modified test protocol [33]. According to Sorichter et al. [33], it has been shown that running down, i.e. eccentric effort, is an effective way to cause such a load on skeletal muscle that it can induce delayed onset muscle soreness (DOMS) symptoms. During the downhill running tests heart rate (HR) and blood lactate concentrations (LA) were measured. All subjects participated in the third laboratory protocol after 3 weeks of vitamin D supplementation or placebo according to the same ExE protocol.

### Measurements and blood collection

At the beginning of the study (pre-intervention) and at the end of each treatment period (post- intervention supplementation or placebo protocol) all subjects reported to the laboratory and had venous blood drawn for the determination of levels of 25(OH)D and muscle biomarker concentrations. The blood samples were collected to determine the aforementioned markers immediately before (rest), immediately after the eccentric exercise (max) and during post-workout recovery (1 h and 24 h after the end of the test). All investigated subjects underwent bioelectric impedance analysis (InBody Data Management System) under resting conditions to determine their body mass.

### Biochemical analyses

For biochemical analysis, antecubital venous blood samples were always drawn at the same time of day, with the subject in a seated position. Blood was allowed to clot at room temperature and then centrifuged. The resulting serum was aliquoted and frozen at -80 °C for later analyses. The measurements of serum 25(OH)D levels were performed using 25OH- Vitamin D ImmunoAssay(DIA source 25OH Vitamin D total RIA CT Kit, Belgium). Intra- and interassay coefficients of variation for 25(OH)D were 5.9–3.3% and 7.4–4.9%, respectively. The measurements of troponin (TN) were performed using Human TNNI1 (Troponin I Type 1, Slow Skeletal ELISA Kit EH-0625, Fine Biological Technology, Co Ltd. Wuhan, China). Intra- and interassay coefficients of variation for TN were < 8.0% and < 10.0%, respectively. The serum myoglobin (MB) levels were measured using Human Myoglobin Enzyme Immunoassay (Mioglobina ELISA, KIT DRG® Myoglobin, EIA-3955). Intra- and interassay coefficients of variation for MB were 3.9–6.6% and 7.8–7.2%, respectively. The lowest detectable level of myoglobin by this assay is estimated to be 5 ng/ml. The proinflammatory cytokines interleukin-6 (IL-6) levels were measured by using Human IL-6 High Sensitive ELISA kit, Diacone, France. Intra- and inter-assay coefficients of variation for of IL-6 were < 4.4% and < 6.4%, respectively and tumor necrosis factor-alpha (TNF-α) were performed using (TNF-α-EASIA KAP1751 firm DIAsource, Belgium). Intra- and interassay coefficients of variation for TNF-α were < 5.1% and < 8.6%, %, respectively.

Creatine Kinase (CK) and Lactate Dehydrogenase (LDH) activity were measured using a commercial kit (CK NAC and LDH P-L, RANDOX, UK). Intra- and interassay coefficients of variation for CK were 2.3–1.5% and 3.9–3.3%, respectively and for LDH were 3.9–1.8% and 4.0–2.8%, respectively. Blood lactate concentrations (LA) were determined using BiosenC_line method (EKF Diagnostic GmbH, Germany). The degree of hemoconcentration (%) was calculated according to formula of subtracting the peak hematocrit with the minimum hematocrit recorded and multiplying by 100; all biochemical variables levels were corrected according to plasma volume.

### Statistical analysis

Shapiro-Wilk, Levene’s and Mauchly’s tests were used in order to verify the normality, homogeneity and sphericity of the sample’s data variances, respectively. The magnitudes of differences between results of pre-test and post-test were expressed as a standardized mean difference (Cohen effect sizes). The criteria to interpret the magnitude of the effect sizes were: < 0.2 trivial, 0.2—0.6 small, 0.6—1.2 moderate, 1.2—2.0 large and > 2.0 very large. Descriptive statistics were calculated and the results were presented as means and standard deviations (mean ± SD). We analyzed differences between pre- and post-intervention (placebo/vitamin D) baseline and post exercise variables. The data were analyzed by two-way ANOVA followed by the Student-Newman-Keuls test when appropriate. The statistical analysis includes a two-way ANOVA (placebo vs. vitamin D) and pre intervention vs. post intervention. Pearson correlation coefficients were analyzed to determine the inter-variable relationships. All analyses were performed using the Statistica v. 12 statistical software package (StatSoft, Tulsa, OK, USA). Statistical significance was set at *p* < 0.05.

## Results

The effects of dietary supplementation with vitamin D and placebo administration on serum 25(OH)D, muscle biomarkers and proinflammatory cytokines concentrations in runners were compared after three weeks of each treatment protocol. Analysis of variance revealed a significant effect of vitamin D supplementation on serum 25(OH)D concentration (F = 17.1; *p* < 0.001). Significant differences between pre-intervention and post-intervention baseline serum 25(OH)D levels (*p* = 0.02) and post-exercise levels were observed in EXP group (*p* < 0.001) (Table 3). A significantly higher post-intervention 25(OH)D level was observed in EXP group compared to placebo CON at rest (40.3 ± 4.9 versus 31.8 ± 4.2 ng/ml, *p* < 0.05, respectively) and during post-workout recovery (*p* < 0.05). The vitamin D increased baseline 25(OH)D (Δ) by 5.4 ± 2.8 ng/ml and decreased placebo by - 2.2 ± 3.6 ng/ml. ANOVA revealed a significant effect of vitamin D diet on TN levels (F = 11.6; *p* < 0.01). A significantly lower post-exercise TN level was observed in EXP group compared to pre-supplementation values (*p* = 0.004) and compared to CON group (*p* < 0.05). A significant effect of vitamin D supplementation was observed in response to MB levels (F = 9.0; *p* < 0.01) and TNFα (F = 4.7; *p* < 0.05). A repeated measure of two-way ANOVA revealed the significance of diet and exercise interaction effects on MB (F = 4.5; *p* < 0.01), CK (F = 4.5; *p* < 0.01) and 25(OH)D concentration (F = 3.2; *p* < 0.05). A significantly lower 1 h post-exercise MB level was observed in EXP after vitamin supplementation (*p* = 0.01). Lower 24 h post-exercise CK activity was observed after vitamin D diet compared to the placebo group (*p* < 0.05). No significant effect of vitamin D diet was observed regarding LDH activity at baseline and at post-exercise levels. Significant lower max and 1 h post-exercise TNFα levels were observed after vitamin D diet compared to pre-intervention (*p* = 0.03 and *p* = 0.02, respectively) and a non-significant trend to lower IL-6 levels. Significant lower 24 h post-exercise IL-6 level was observed in EXP group compared CON in response to interventions (Table 3).

**Fig. 1:**
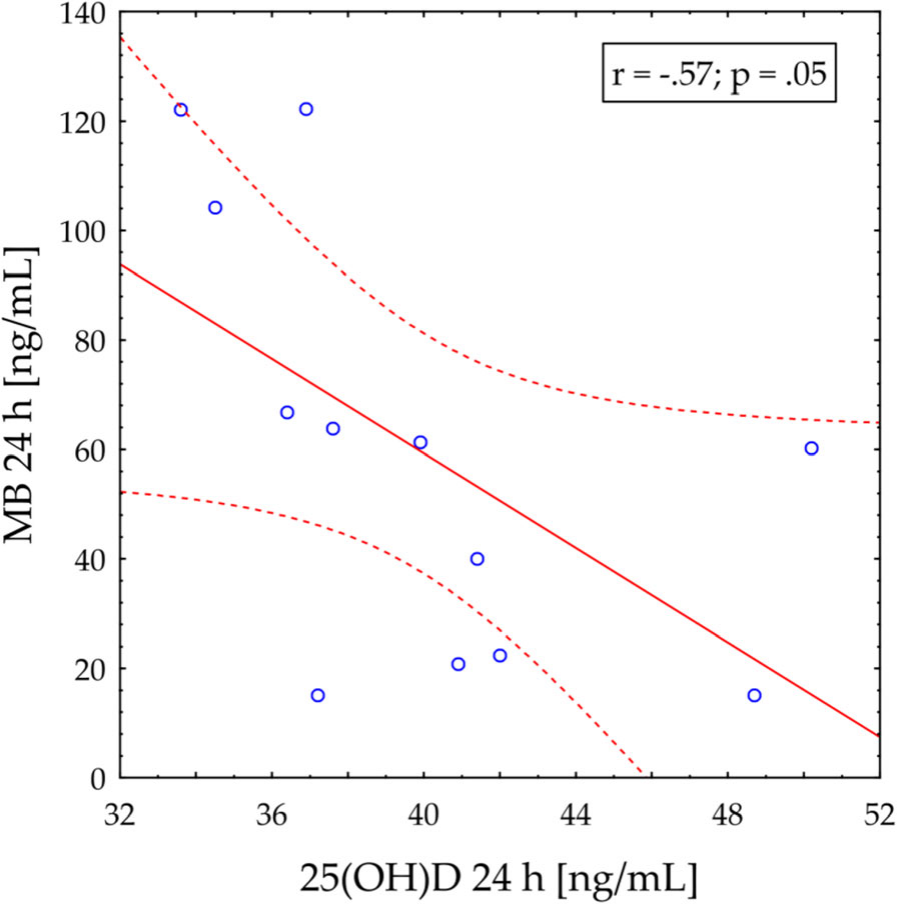
Correlation between 25(OH)D concentration and myoglobin (MB) level (24 h post ExE) in vitamin D supplemented group

**Fig. 2:**
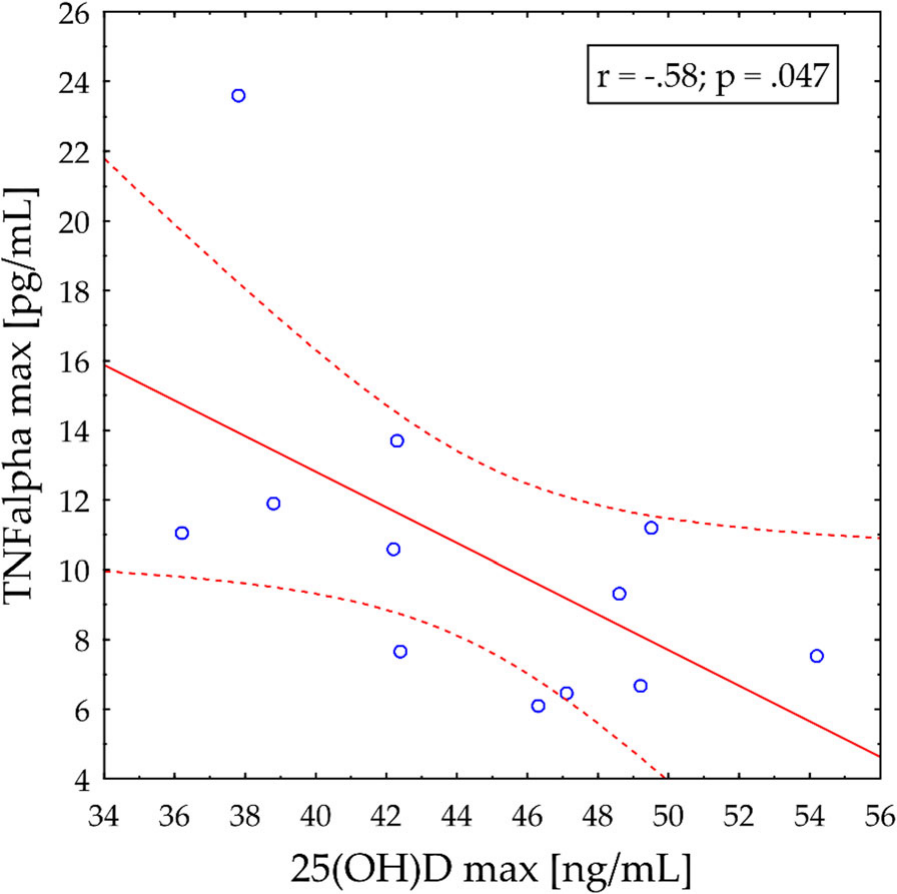
Correlation between 25(OH)D concentration and TN alpha level (24 h post ExE) in vitamin D supplemented group

A significant and negative correlation was observed between 25(OH)D concentration and 24 h post-exercise MB concentration (r = − 0.57; *p* = 0.05) in EXP group (Fig. 1). Importantly, a negative correlation was observed between 25(OH)D concentration and TNFα levels (r = − 0.58; *p* < 0.05) (Fig. 2) only in vitamin D supplemented group. ANOVA did not reveal any significant effect of diet on HRmax (157.0 ± 5.0 versus 154.0 ± 3.0 b/min) and serum LA (1.9 ± 0.3 versus 1.8 ± 0.3 mmol/L) concentrations in response to ExE (*p* > 0.05).

## Discussion

The present study was undertaken to investigate whether vitamin D supplementation may modify association between serum 25(OH)D concentrations and skeletal muscle biomarkers, and amplify recovery mechanisms in marathon runners. Our results have demonstrated that a three-week low dosage of vitamin D supplementation caused an elevation of baseline serum 25(OH)D compared to pre-supplementation levels. Significant higher serum 25(OH)D levels were also observed in the vitamin D supplemented (EXP) group compared to the placebo control group. Moreover, the increased 25(OH)D production seemed to have a significant effect on resting and eccentric exercise–induced skeletal biomarker levels and proinflammatory cytokines. The major findings of our study are that a greater 25(OH)D expression in response to vitamin D diet negatively correlated with biomarkers of skeletal muscle damage and that this effect was more pronounced during 24 h recovery period. Three weeks of supplementation had a beneficial effect on certain parameters of skeletal muscle function. Lower serum levels of biomarkers of skeletal muscle damage and vitamin D status improvement might, in turn, have significantly decreased the individual recovery time from eccentric exercise. The study design is unique in that only subjects with serum 25(OH)D levels above 30 ng/mL were included in order to minimize confounding by underlying vitamin D deficiency.

Data concerning positive impacts of vitamin D consumption on optimizing athletic performance and recovery in intensely trained athletes are still sparse [12, 21, 34]. Most studies supported the benefits of dietary supplementation with vitamin D in healthy untrained adults and people diagnosed with 25-hydroxyvitamin D insufficiency (< 30 ng/ml) [25, 35, 36]. Contrarily, a recent meta-analysis involving 532 athletes found no improvement in measures of physical performance despite the inclusion of vitamin D deficient athletes at baseline and improvements in vitamin D levels over mean 12 weeks of follow-up [5].

Previous results revealed a positive effect of vitamin D supplementation on global muscle strength, power and muscle fatigue risk factors [15, 16, 18, 37]. The effectiveness of the vitamin D supplementation was confirmed in athletes, however, the optimal intake and serum 25(OH)D levels have yet to be identified in the athletic population [2, 3].

It has been suggested that different muscle groups and muscle contraction may respond differently to vitamin D supplementation [31, 38, 39]. In the study of Zhang et al. [38], vitamin D supplementation positively affected lower limb muscle strength, but not muscle power in athletes. Significant improvements in muscle function following vitamin D repletion were reported in response to high intensity exercise [40]. These results differ from those of Nieman et al. [31], who showed that a large-dose vitamin D_2_ supplementation in athletes during a 6-week period had no effect on total 25(OH)D and muscle function.

A significant and positive correlation was observed between 25(OH)D levels and aerobic performance (VO_2_max) and training status [41, 42]. Supplementation with a supraphysiological dose of vitamin D (6000 IU/day) during a 8-week period of training in rowers with sufficient 25(OH)D levels significantly increased VO_2_max compared to a placebo group [26]. However, no significant effects of vitamin D on athletic performance or a potential association between 25(OH)D levels and an individual’s VO_2_max were also noted [42, 43].

The main strength of our study was the inclusion of serum 25(OH)D concentrations in examining the muscle damage response in endurance training runners. Several mechanisms have been reported that may be responsible for the protective and ergogenic effect of 25-hydroxycholecalciferol in skeletal muscle [15]. The proposed mechanisms include a role of vitamin D receptors (VDR) that are expressed in skeletal muscle and when bound to 1,25(OH)_2_D_3_, exert genomic effects at target sites [25]. Another mechanism includes a role of supplementation with vitamin D in stimulating oxygen uptake in skeletal muscle. It has been hypothesized that positive effects of 25(OH)D on oxygen uptake could be due to the fact that the cytochrome enzymes activating vitamin D into 1,25dihydroxycholecalciferol have heme-containing proteins that could potentially affect the binding affinity of oxygen to hemoglobin [44]. A significant effect of both exercise training and vitamin D supplementation on increased force and power output of skeletal muscle perhaps in response to an enhanced cross-bridge cycling and muscular contraction has also been suggested [23, 45, 46].

Strenuous exercise with eccentric muscle contractions may be attributed to muscle fatigue due to muscle membrane damage along with increase intermuscular enzymes and biomarkers [2, 9, 31, 32, 47]. In our study, we concluded that 25(OH)D production after vitamin D supplementation has a significant effect on selected biomarkers of skeletal muscle damage and post-exercise proinflammatory cytokine levels. A significant and negative correlation was observed between 25(OH)D and MB concentration in response to a vitamin D diet. Importantly, a negative correlation was observed between 25(OH)D concentration and TNFα levels during the 24 h recovery period. This support the findings that lower serum levels of biomarkers of skeletal muscle damage and vitamin D status improvement, might, in turn, have significantly decreased the individual recovery time in marathon runners participating in mountain events [2]. Lower levels of serum vitamin D have been associated with increased muscle weakness, fatigue and injury incidents [48]. Therefore, the ability to reduce fatigue and decrease the recovery time is important for athletes who train at high and moderate intensity with both concentric and eccentric muscle contraction more frequently [2, 49].

It was also observed that during recovery 1,25-hydroxyvitamin D increases the myogenic differentiation and proliferation, down-regulates myostatin and improved the skeletal muscle regeneration in animal studies [18]. The findings that vitamin D supplementation enhances the recovery process following intense exercise [20] and ultramarathon runs [50] were also supported by human studies. Serum 25(OH)D concentrations correlated positively with physical activity scores, and negatively with body mass index, lipid profile, fatigue scores (visual analog scale), and muscle fatigue biomarkers in healthy older adults [51, 52]. Higher 25(OH)D levels were accompanied by lower creatine kinase, troponin I, and lactic acid dehydrogenase activity, the generally used biomarkers for earlier detection of muscle injury, especially muscle soreness following training interventions [35]. In the study of Nowak et al., self-reported fatigue has been linked to low levels of circulating 25-hydroxyvitamin D (25OHD), a biomarker of vitamin D status, however, vitamin D treatment significantly improved fatigue in healthy persons with vitamin D deficiency [53].

Fatigue is a complex and non-specific phenomenon with significant response to physical and mental exertion or a feature of illnesses. There is no generally accepted set of criteria for fatigue, and the prevalence of fatigue varies widely depending on the assessment method [54–57]. A previous study demonstrated that vitamin D supplementation attenuated the inflammatory biomarkers immediately following intensive exercise with both eccentric and concentric muscle contractions [20]. Our results revealed lower post-exercise TNF-α levels and a tendency towards lower IL-6 concentrations in a specifically trained supplementation group compared to the baseline levels. Regardless of the fact that long-term exercise training might diminish 25(OH)D concentrations, we conclude that a dietary vitamin D supplementation also has a beneficial effect on the function of the immune system by suppressing exercise-induced proinflammatory cytokines in elite athletes. Still, a question arises whether the recommended dosage of 1500–2000 IU/day vitamin D could maintain adequate serum vitamin D concentrations in endurance trained athletes. The optimal levels needed for athletic performance are controversial; lower than 1000 UI/day may not be sufficient, especially for an older athletic population. It has been shown that dosages higher than 2000 UI/day or 3000 UI/day have been sufficient to increase skeletal muscle function and reduce the risk of stress fractures [24, 56, 57]. These preliminary findings highlight the requirement for further studies on the effects of different dosages of vitamin D supplementation on skeletal muscle function and optimal performance in athletes.

## Conclusions

Three weeks of vitamin D supplementation had a positive effect on serum 25(OH)D levels in endurance trained runners and it caused a marked decrease in post-exercise biomarkers levels. We concluded that vitamin D supplementation might play an important role in the improvement of muscle function and prevention of skeletal muscle injuries following exercise with eccentric muscle contraction in athletes.

## Data Availability

Upon request from the first author.
